# Safe but broken: a critical review on psychological risks of childhood in refugee camps

**DOI:** 10.3389/fpsyt.2026.1763375

**Published:** 2026-03-12

**Authors:** Sandra Figueiredo, Genta Kulari

**Affiliations:** Researcher of University Research Centre in Psychology (CUIP), Department of Psychology, Universidade Autónoma de Lisboa Luís de Camões, Lisbon, Portugal

**Keywords:** camps, ecological model, meta-synthesis, PTSD, refugee children, urban resettlement

## Abstract

The concept of severely deprived children has recently been integrated into the study of refugee children. While refugee camps are designed to ensure physical safety, they often expose young residents to chronic deprivation, limited mobility, and psychosocial isolation. Conversely, urban resettlement may foster autonomy and integration yet introduce new forms of structural and cultural stress. Understanding how environmental context shapes trauma and coping among refugee youth is essential to designing context-sensitive interventions. A qualitative brief meta-synthesis was previously conducted with 984 refugees following the five-stage approach proposed by Lachal et al. Twenty-four peer-reviewed qualitative and mixed-methods studies published between 2017 and 2025 were retrieved from PsycINFO, PubMed/Medline, Scopus, CINAHL, and Web of Science. The synthesis was guided by Bronfenbrenner’s ecological model to capture multilevel environmental influences on mental health. Across both contexts, post-traumatic stress disorder (PTSD), depression, anxiety, and somatization were consistently prevalent, though their presentation differed. In four refugee camps (248 individuals), cumulative trauma exposure, legal uncertainty, and spatial confinement intensified acute distress and collective grief, often manifesting through somatic symptoms and perceived helplessness. In 19 urban resettlement cases (736 individuals, 677 being children), refugees displayed lower acute stress but higher chronic depression and adjustment difficulties, largely driven by discrimination, social isolation, and integration. Narratives emphasized hope, agency, and bicultural adaptation as key resilience mechanisms. Environmental context fundamentally shapes refugee children’s psychological trajectories: camps amplify survival-based distress, whereas resettlement introduces persistent psychosocial strain. Policies must integrate trauma-informed and culturally responsive interventions that address both confinement-related and integration-related stressors.

## 1 Introduction

Since 2018, more than 11 million refugees have been children. These individuals have faced two major challenges that disrupt their lives and developmental trajectories: they have either been allocated to refugee camps or relocated to host countries to initiate the resettlement process (1–3). Refugee camps were originally established to provide temporary shelter for displaced persons and to mitigate disruptions in host societies (4, 5). However, many refugees remain in these camps long term, depending on host-country policies and restrictions. Over the past decade, limited progress has been made by individual nations and the United Nations High Commissioner for Refugees (6) to improve living conditions and to counteract negative stereotypes associated with camp life (7, 8).

Residence in a refugee camp has been described as an incarceration-like experience that can exacerbate trauma (3, 9). This dual burden is particularly detrimental for children and adolescents, as they are exposed to two intersecting forms of trauma: the trauma of forced displacement due to war or other human-rights violations, and the trauma associated with living in camps characterized by restrictive conditions, limited mental-health support, and minimal social and primary resources (10–12). Systematic reviews conducted up to 2021 indicate that there has been little advancement in qualitative research examining how trauma and posttraumatic stress disorder (PTSD) manifest among children living in harsh, non-humanitarian conditions in refugee camps (13–17). Unaccompanied minors represent a particularly vulnerable group and have recently been classified as severely deprived children, facing heightened risks of sexual exploitation within camps, an area for which research remains critically insufficient (18–20). Regarding the concept of severely deprived children, this term refers to children who experience profound deficits in basic needs, including nutrition, shelter, education, and emotional care. Such deprivation can have long-lasting effects on their physical, cognitive, and socio-emotional development. These challenges are often more severe for children with special education needs, particularly when parents experience stress-related difficulties, which may be further exacerbated by forced mobility (21). Refugee and displaced children are especially vulnerable to severe deprivation, underscoring the need for targeted interventions and protective measures to support their well-being.

Since 2018, the escalation of war conflicts has intensified pressure on host countries, particularly in Europe, where infrastructure and resources are often inadequate to accommodate growing refugee populations (6). Urban integration, wherein refugees reside outside camps and are absorbed into host communities, represents an alternative yet still vulnerable context. Compared with camps, urban resettlement is generally perceived more positively by refugees (22–24). However, refugee children typically encounter new challenges, including navigating cultural and religious differences between their country of origin and their host country (25, 26). Practitioners involved in social, educational, and psychoeducational interventions must prioritize culturally sensitive approaches to mitigate these challenges (27, 28). Without adequate support, refugee youth are at heightened risk of social isolation, which can intensify their sense of loss and loneliness. There are coping mechanisms that can be addressed, for example, the supportive role of pets is often underestimated in these populations, particularly in relation to mental health and healthcare provision. Pets can influence health behaviors, emotional well-being, and engagement with healthcare services, particularly during resettlement in a new country. Healthcare providers who acknowledge the presence of companion animals may better understand patients’ coping strategies, sources of comfort, and practical barriers to care, such as housing restrictions, access to veterinary services, or difficulties attending appointments due to pet care responsibilities (29). To add, school dropout, and housing insecurity, which may exacerbate negative acculturation outcomes such as intrusive memories, depressive symptoms, and prolonged distress. Therefore, while urban resettlement addresses some structural limitations of camps, it introduces new, culturally mediated stressors that can increase trauma and PTSD risk. Cultivating a sense of belonging among refugee youth is paramount to preventing trauma escalation, and early PTSD screening for both children and caregivers is critical during the initial stages of resettlement and education (21).

From an ecological perspective, distinguishing between camp-based and urban resettlement contexts is essential for understanding trauma development in forcibly displaced children and adolescents (30–32). Bronfenbrenner’s ecological model provides a comprehensive framework for analyzing the interplay between environmental, social, and individual factors in shaping refugee mental health (33–35). At the microsystem level, urban integration exposes refugees to immediate environments including family, peers, schools, workplaces, and local communities (36). Evidence from Germany, Norway, and Sweden (37, 38) demonstrates that community-dwelling refugees experience persistent depression, anxiety, and PTSD symptoms, often linked to daily social interactions and available support networks. Unlike camp settings, where social interactions are structured yet limited, urban environments present both opportunities for support and challenges, such as social isolation, discrimination, and restricted access to culturally sensitive services (39). These microsystemic interactions critically influence coping strategies, resilience, and help-seeking behaviors, as measured by instruments such as the HSCL-25, WHO-5, and Refugee Health Screeners (29, 40).

The mesosystem refers to the interconnections among microsystems, including family–school–community linkages and interactions with healthcare providers and local organizations. Evidence from the Netherlands and Germany (41, 42) indicates that refugees’ mental-health outcomes are strongly influenced by the accessibility and coordination of services across these systems. Robust mesosystemic integration—through collaborative approaches between schools, social services, and mental-health providers—can mitigate chronic PTSD and depression. Conversely, fragmented mesosystem interactions exacerbate stress, particularly for children and adolescents who depend on structured support for psychosocial adjustment.

At the exosystem level, broader societal structures, including government policies, urban planning, and social-welfare provisions, indirectly affect refugees’ psychological well-being. Urban-resettled refugees are more exposed to these exosystemic influences than camp residents. Studies from Germany, Sweden, and Norway (43–45) demonstrate that employment opportunities, housing stability, and legal status significantly impact stress, depression, and anxiety. The exosystem also mediates access to mental-health services, with disparities observed in the availability of psychosocial interventions and community-based programs across host countries.

The macrosystem, encompassing cultural values, societal norms, and host-country attitudes toward refugees, further shapes mental-health outcomes. Research across multiple European contexts (46, 47) shows that inclusive societal attitudes, anti-discrimination policies, and culturally responsive mental-health services enhance resilience, whereas xenophobia and systemic barriers exacerbate PTSD, depression, and somatization. These macrosystemic factors interact dynamically with the microsystem and mesosystem, influencing daily experiences of acceptance, social integration, and self-efficacy.

Finally, the chronosystem emphasizes temporal dynamics, including the timing of displacement, duration of urban integration, and historical context of migration policies. Longitudinal evidence from Norway and Germany (38, 42) suggests that prolonged exposure to post-migration stressors—such as unemployment, housing instability, or social isolation—can intensify trauma over time, reinforcing chronic mental-health conditions. Early interventions during initial stages of urban integration may prevent the consolidation of PTSD and depressive disorders, highlighting the importance of temporally sensitive support strategies.

The present study examines differences among refugees (children and adolescents up to 19 years old) living in camps versus host urban communities in Europe, focusing on mental-health challenges and the ways in which refugee youth themselves perceive and report these experiences across the two settings after forced mobility.

## 2 Method

### 2.1 Research design

We previously developed a systematic critical review consisting of a qualitative meta-synthesis completed in 2025 (Kulari & Figueiredo, 2025 (45)—PROSPERO: https://www.crd.york.ac.uk/PROSPERO/view/CRD420251153819) to determine outcomes from experiences of 984 refugees (736 individuals in urban areas, 677 being children), in camps 65 are children (up to 11 years old) and 612 are adolescents; the remaining refers to parents and other adults in the sample of each study) after forced displacement and resettlement in different hosting countries. Regarding sex, only one study exclusively included female participants (188 Syrian girls). None of the included studies explicitly reported a boys-only sample. The remaining studies did not provide sex-disaggregated data, limiting comparative analysis by sex.

While this study builds upon a previously published meta-synthesis by the same authors, the present work introduces an original analysis focused on differences between refugees living in camps and those living in urban settings, which was not addressed in the earlier publication.

The included camp-based studies collectively illustrate that refugee children’s psychological experiences are profoundly shaped by the material deprivation, social isolation, and uncertainty inherent in prolonged displacement. Across diverse contexts, common patterns emerge: children exhibit heightened stress, limited access to educational and psychosocial resources, and vulnerability to both acute and chronic trauma. These findings suggest that the socio-political structures of camps—not only individual circumstances—play a decisive role in shaping mental health outcomes, highlighting the need for interventions that address both environmental conditions and individual support.

The critical appraisal methods were followed during the review that underpinned the meta-synthesis. We conducted this meta-synthesis following five consecutive stages: (1) determination of the focus of the review; (2) assessment of selected articles in terms of research quality; (3) identification and extraction of relevant data and abstraction of key themes in each article; (4) comparison and synthesis of the key themes to generate new meaning; and (5) examination of the validity of the analysis and interpretation of the results (48).

### 2.2 Search strategy

The search strategy combined controlled vocabulary and free-text terms related to refugee populations, age groups, and mental health outcomes. Boolean operators were applied as follows: (“refugee*” OR “asylum seeker*” OR “forced migrant*”) AND (“child*” OR “adolescent*” OR “youth” OR “minor*”) AND (“trauma” OR “posttraumatic stress disorder” OR “anxiety” OR “depression” OR “mental health” OR “psychosocial wellbeing”) AND (“refugee camp*” OR “settlement*” OR “resettlement” OR “urban setting*”). Search strings were adapted to the syntax and indexing terms of each database.

### 2.3 Study selection and quality assessment

A PRISMA flow diagram illustrating the identification, screening, eligibility, and inclusion phases is presented in the previous work (45). We followed a stepwise process in the selection of studies. First, we searched databases and specific journals. We retrieved 1,859 records after the initial search. After reading abstracts, we re-evaluated eligibility. We excluded duplicates (n = 1,220) and irrelevant articles based on the inclusion and exclusion criteria (n = 251). In the second step, we examined the full texts of the 388 retained articles and removed 366 that did not fit the research question; we also added two articles from reference lists. We appraised article quality according to the criteria recommended by Atkins et al. (49). Finally, we retained 24 articles in this meta-synthesis for further analysis.

We assessed the quality of included articles using the Critical Appraisal Skills Programme (CASP 2018) checklist for qualitative studies. The CASP consists of 10 questions to evaluate systematically selected articles; these questions were answered “yes” or “no.” Two reviewers independently assessed study quality. Disagreements were resolved through discussion. No studies were excluded based solely on quality appraisal; however, methodological rigor was considered during the interpretation and weighting of findings in the synthesis. Sample questions include “Was there a clear statement of the aims of the research?” and “Is a qualitative methodology appropriate?”

## 3 Results

Across the 24 reviewed studies, PTSD, depression, anxiety, and somatization consistently emerged as dominant psychopathological outcomes among refugee populations, although their prevalence and phenomenology varied according to the environment of displacement. Within camp settings, findings suggest that children and adolescents frequently display heightened psychological symptomatology, driven by the cumulative interaction of pre-migration trauma exposure and ongoing environmental deprivation (50–53). Expression of distress among refugees immersed in camps (5 of the 24 studies) was lower in verbal expressivity and higher in somatic or nonverbal manifestations compared with urban resettlement settings. Examples include prolonged stays in Jordan (Azraq Camp) with Syrian adolescents aged 10–17; a migrant detention center in Canada; cohorts in Australia with female adolescents from various origins (21); and Syrian female adolescents in Lebanon, many of whom were married (ages 12–17). All camp or informal accommodation settings are expected to be temporary but were verified as involving extended periods of settlement and confinement, adversely affecting mental health and PTSD development (54–56).

The camp context—typically characterized by legal uncertainty, restricted mobility, overcrowding, and chronic resource scarcity—amplifies stress reactivity and limits opportunities for psychosocial recovery, affecting a total of 248 refugees living in camps across the reviewed studies. Studies employing diagnostic tools such as the Harvard Trauma Questionnaire (HTQ) and the Hopkins Symptom Checklist (HSCL-25) document high rates of PTSD and comorbid depression among displaced populations living under constrained conditions (57–59). Notably, the embodiment of distress is salient in these contexts: somatic manifestations such as gastrointestinal complaints, headaches, and sleep disruptions (mainly when sleep routines are affected in terms of cosleeping) are prevalent and often constitute culturally mediated expressions of psychological suffering (5, 42, 60). This psychosomatic dimension underscores how prolonged exposure to environmental stressors in camps transforms trauma into persistent physiological dysregulation. See **Tables 1** and **2**.

**Table 1:** Refugee children (248) in camps and respective experiences.

Study	Hosting country	Participants	Primary objective
Kronick et al. (2018) (50)	Canada	35 children (20 families) in a detention center	Understand the experiences of children in migrant detention centers.
Roupetz et al. (2020) (61)	Lebanon	118 Syrian girls (ages 12–17)	Examine experiences and challenges of Syrian refugee girls, including education, safety, and social support.
Sajdi et al. (2021) (62)	Jordan	15 adolescents (ages 10–17) in Azraq camp	Explore experiences of Syrian adolescents in refugee camps regarding education, bodily integrity, and psychological well-being.
Al-Shatanawi et al. (2023) (54)	Jordan	80 participants (health professionals, teachers, parents, and adolescents)	Assess psychosocial problems and coping mechanisms of Syrian refugee adolescents.

The complete list of references appears in the previous published work (45).

**Table 2:** Refugee children (N = 736) resettled in urban areas and respective experiences.

Study	Hosting country	Participants	Primary objective
DeClercq et al. (2025) (63)	USA	68 Syrian youth (ages 7–17)	Analyze linguistic elements in traumatic narratives and psychosocial correlates.
Khawaja & Schweitzer (2024) (18)	Australia	19 (ages 15–18, from 12 countries)	Explore identity and experiences of young refugees in the context of resettlement.
Jarbly et al. (2018) (64)	Denmark	7 (ages 17–18 from Middle East and Southest Asia	Explore unaccompanied refugee adolescent’s perspective on healing and the mental healthcare offered to them when resettled
Magan et al. (2024) (55)	USA	15 Rohingya adolescents (ages 12–17)	Explore emotional and mental health challenges among Rohingya adolescents in resettlement.
Filler et al. (2021) (65)	Canada	7 adolescents and 8 service providers	Investigate how Syrian adolescents conceptualize mental health and the role of services.
Kwon & Lee (2018) (66)	South Korea	4 North Korean children (ages 8–9)	Understand traumatic experiences and resilience processes via play-based group therapy.
Gušić et al. (2018) (67)	Sweden	40 adolescents (ages 13–19)	Explore dissociative experiences in war-traumatized refugee youth.
Hettich & Meurs (2020) (68)	Germany	1 Afghan youth (age 18)	Analyze perceptions of psychosocial support received after arrival in the host country.
Majumder et al. (2019) (56)	UK	15 adolescents (ages 15–18)	Examine unaccompanied refugee minors’ perceptions of mental health services.
Yetim et al. (2025) (69)	Turkey	24 Syrian youth (ages 12–18)	Investigate stress factors and coping processes of newly resettled Syrian youth.
Abdelhamid et al. (2023) (70)	Germany	47 parents and 11 children (ages 8–17)	Explore refugee families’ perceptions of factors affecting child well-being.
d’Abreu et al. (2020) (71)	USA	7 Syrian adolescents and 4 parents	Analyze acculturation, mental health, and school experiences of Syrian adolescents.
Sleijpen et al. (2017) (72)	Netherlands	21 refugee youth (ages 13–20) with PTSD	Identify factors promoting resilience among refugee youth.
Oztabak (2020) (73)	Turkey	50 children (ages 6–10)	Compare drawings of refugee and non-refugee children regarding war and migration.
Bründlmayer et al. (2025) (35)	Austria	28 African youth (under 18)	Explore narratives and specific needs of unaccompanied refugee minors.
Mattelin et al. (2024) (74)	Sweden	18 youth (ages 15–17) from 12 origins	Describe common experiences and challenges in migration and resettlement.
Moleiro & Roberto (2021) (75)	Portugal	137 unaccompanied minors (<18)	Characterize transition to adulthood and social integration processes.
Majumder (2019) (56)	UK	15 adolescents (ages 15–18)	Explore perceptions and beliefs about mental illness, focusing on stigma.
Santinho & Krysanova (2024) (76)	Portugal	9 adolescents (ages 15–18)	Investigate barriers to inclusion and the role of art as a tool for expression and integration.
Davidson et al. (2021) (77)	Lebanon	188 Syrian girls (ages 13–17)	Describe unmet needs and aspirations of displaced Syrian adolescents.

The complete list of references appears in the previous published work (45).

Conversely, in urban and community-based resettlement contexts, refugee children and youth—particularly those up to 19 years—tend to exhibit lower acute stress reactivity but sustained psychological vulnerability. Evidence from large-scale European studies indicates that community-dwelling refugees experience ongoing depressive symptomatology, generalized anxiety, and functional impairment, as measured by instruments such as the WHO-5 Well-Being Index, Patient Health Questionnaire (PHQ-9), and Kessler Psychological Distress Scale (K10) (37, 38, 42). These outcomes are dynamically shaped by post-migration stressors—language barriers, unemployment, social isolation, and experiences of discrimination (40, 45, 65). The persistence of these psychosocial burdens, despite the relative material advantages of urban life, reveals that integration processes themselves can be sources of chronic stress.

When contrasting both contexts, PTSD emerges as a trans-situational outcome, pervasive across settings yet qualitatively distinct in its expression. Camp-based youth display acute trauma symptomatology closely tied to prior exposure and ongoing insecurity, while community-based counterparts present moderated PTSD profiles, in which symptom severity is influenced by social support networks and opportunities for integration (15, 42). Depression and anxiety are prevalent in both environments but follow different etiological pathways: in camps, they are reactive responses to immediate stress and loss, whereas in urban environments, they stem from long-term adjustment challenges and identity negotiation (78–80). Somatization is disproportionately represented among camp residents (42, 60), reflecting both limited healthcare access and the psychophysiological embodiment of chronic adversity. In contrast, functional and social impairment are more prominent among community-based refugees, where unemployment, cultural adaptation barriers, and fragile social networks contribute to diminished quality of life and perceived social marginalization (38, 42, 45). Protective factors such as social support, host-language proficiency, and targeted psychosocial interventions are more prevalent in community settings, partially mitigating adverse mental-health trajectories (45, 63). Within camps, the limited availability of structured psychosocial programs leaves these mechanisms underdeveloped, emphasizing the need for context-sensitive interventions that balance protection with empowerment.

## 4 Discussion

The present meta-synthesis provides empirical support for Bronfenbrenner’s ecological model by demonstrating that refugee children’s and adolescents’ mental health outcomes are shaped by continuous interactions between individual trauma exposure and multilayered post-migration environments (33, 58). Rather than representing static consequences of war-related experiences, psychological difficulties emerge dynamically from the social, institutional, and structural contexts in which displaced youth attempt to rebuild their lives. The findings demonstrate that psychological distress among refugee children and adolescents is not solely determined by pre-migration trauma exposure, but is dynamically shaped by multilayered post-migration environments, including camp-based confinement and urban resettlement conditions.

At the microsystem level, daily interactions with family members, peers, schools, and immediate community environments strongly influenced emotional regulation, coping strategies, and help-seeking behaviors. In camp settings, restricted mobility, overcrowding, and limited access to education and recreational activities constrained children’s opportunities for developmental recovery, reinforcing trauma-related symptoms and somatic distress. In contrast, urban resettlement contexts offered greater educational and social opportunities but simultaneously exposed youth to discrimination, linguistic barriers, and social exclusion, which contributed to chronic depressive symptoms and anxiety. These findings support previous ecological models of refugee distress that emphasize the importance of daily stressors in shaping post-traumatic outcomes (58).

At the mesosystem level, the quality of connections between families, schools, social services, and healthcare providers emerged as a central protective factor. Studies included in showed that coordinated institutional support facilitated resilience, school engagement, and psychosocial stabilization, whereas fragmented service delivery exacerbated vulnerability. This reinforces Bronfenbrenner’s proposition that developmental outcomes depend not only on isolated environments, but on the strength of intersystem connections.

Exosystem influences, such as asylum policies, housing stability, access to welfare systems, and labor market integration, were particularly salient in urban resettlement contexts. Legal insecurity, unstable housing, and limited access to culturally sensitive mental health services indirectly increased psychological burden among refugee families and youth. At the macrosystem level, societal attitudes toward refugees, including experiences of xenophobia and discrimination, shaped children’s sense of belonging and identity development. Inclusive policies and culturally responsive educational environments were associated with improved psychosocial adjustment, whereas hostile social climates intensified distress and social withdrawal.

Finally, the chronosystem dimension highlights how prolonged exposure to post-migration stressors contributes to the chronicity of psychological symptoms. Children residing long-term in camps experienced cumulative trauma effects, while those in urban contexts often faced persistent integration-related stress that gradually transformed acute trauma reactions into long-term depressive and anxiety disorders. This temporal perspective underscores the importance of early intervention during critical transitional periods, such as initial resettlement and school integration.

Therefore, at the level of proximal environments, daily interactions within families, schools, peer groups, and immediate community settings played a central role in shaping emotional regulation and coping processes. In camp-based contexts, restricted mobility, overcrowding, and limited access to education and structured activities reinforced survival-oriented stress responses and contributed to heightened PTSD symptoms and somatic expressions of distress, consistent with previous evidence on camp-related deprivation and trauma chronicity (9, 40). In contrast, urban resettlement contexts offered greater access to educational opportunities and social participation but simultaneously exposed refugee youth to discrimination, language barriers, and social isolation, which were associated with persistent depressive symptoms, anxiety, and functional impairment, as reported in earlier systematic reviews (8–12).

The quality of interconnections between educational, healthcare, and social support systems further influenced mental health trajectories. Coordinated institutional responses facilitated psychosocial adjustment and resilience, whereas fragmented service provision limited access to early intervention and prolonged psychological vulnerability. These findings reinforce the ecological assumption that mesosystemic integration plays a critical role in shaping developmental and mental health outcomes (33).

Considering comparison between systematic reviews that served the purposed of this review, the present findings are largely consistent with previous systematic reviews documenting high prevalence rates of PTSD, depression, and anxiety among refugee children (45, 63, 78). However, this meta-synthesis contributes novel insights by qualitatively differentiating how these mental health outcomes manifest across environmental contexts. While prior reviews have primarily focused on prevalence estimates and individual risk factors, the current study highlights context-specific symptom expression patterns.

Consistent with recent evidence (8, 40) camp-based studies revealed elevated PTSD symptom severity and high levels of somatization, reflecting the impact of prolonged confinement, material deprivation, and environmental insecurity. Similarly, earlier research has shown that displacement settings characterized by limited autonomy amplify stress reactivity and psychosomatic symptoms. In urban resettlement contexts, the present synthesis emphasized the role of post-migration stressors in sustaining psychological vulnerability. However, this study extends previous work by showing that although acute PTSD symptoms tend to be less pronounced in community settings, refugee youth frequently experience persistent depression, identity-related stress, and functional impairment linked to social exclusion and acculturation challenges.

Moreover, previous meta-analyses have often treated refugee children as a relatively homogeneous group. In contrast, this review underscores the heterogeneity of refugee experiences and highlights the importance of environmental context as a moderating factor in mental health trajectories. By applying an ecological framework, the present study advances a more nuanced understanding of refugee children’s psychological adaptation processes.

## 5 Conclusion

Within camps, the limited availability of structured psychosocial programs leaves these mechanisms underdeveloped, emphasizing the need for context-sensitive interventions that balance protection with empowerment. Although the psychological consequences of forced displacement are evident across all refugee settings, this meta-synthesis demonstrates that the environment of post-migration resettlement fundamentally shapes how trauma, resilience, and coping mechanisms emerge among children and adolescents. Evidence from the 24 analyzed studies indicates that refugee camps—marked by prolonged confinement, overcrowding, limited mobility, and resource scarcity—intensify acute psychological distress, perpetuate somatic forms of suffering, and restrict access to protective social structures. These contexts produce a survival-based stress ecology in which trauma is continuously reinforced by environmental instability.

Conversely, urban resettlement contexts offer comparatively greater autonomy, educational access, and opportunities for social participation. Yet these environments introduce new forms of chronic, structurally embedded stress: discrimination, unemployment, cultural dissonance, and social isolation (38, 42). Refugee youth in community settings frequently demonstrate moderated PTSD profiles but heightened rates of depression and anxiety driven by prolonged integration challenges, identity negotiation, and acculturative pressure.

Across both contexts, several cross-cutting themes emerge:

1. PTSD is pervasive but phenomenologically distinct depending on the context of resettlement;
2. somatization is prominent in camp settings, whereas functional impairment dominates urban samples;
3. resilience is strongly mediated by social support, access to mental-health services, and opportunities for meaningful participation;
4. environmental constraints shape the trajectory of psychopathology, influencing whether distress remains acute or becomes chronic.

These findings underscore the need for interventions that are both trauma-informed and context-responsive. In camps, priority should be given to reducing environmental deprivation, expanding mobility, and introducing structured psychosocial programs tailored to children and adolescents. In urban environments, services must address integration-related barriers, combat discrimination, and strengthen culturally sensitive mental-health responses. Policymakers and practitioners must therefore consider the ecological layers that shape refugee children’s experiences, acknowledging that trauma does not occur in isolation but is continuously shaped by the sociocultural and structural environments in which refugees attempt to rebuild their lives.

## 6 Limitations

Several limitations should be acknowledged. First, publication bias may have influenced the findings, as only peer-reviewed studies were included. This may have excluded relevant qualitative data from grey literature, humanitarian reports, and non-academic sources, which often document refugee experiences in underrepresented settings. Second, the review was restricted to studies published in English, which likely resulted in the exclusion of relevant research conducted in non-English-speaking regions. This language restriction may have contributed to an overrepresentation of studies from Europe and North America, limiting the global generalizability of the findings. Third, there was a marked imbalance between camp-based studies (n = 5) and urban resettlement studies (n = 19). This asymmetry reflects broader research trends but restricts direct comparability between contexts. Consequently, conclusions regarding camp environments should be interpreted cautiously, as fewer empirical sources were available.

Fourth, substantial heterogeneity was observed across included studies in terms of methodological design, participant age ranges, cultural backgrounds, data collection methods, and outcome measures. While this diversity enriches qualitative synthesis, it also limits the possibility of drawing uniform conclusions and highlights the need for more standardized qualitative reporting frameworks in refugee mental health research. Finally, geographic concentration of evidence represents another limitation. Most urban resettlement studies were conducted in European host countries, whereas camp-based research was largely concentrated in Middle Eastern contexts. This uneven distribution restricts the applicability of findings to other regions, such as Sub-Saharan Africa, South Asia, and Latin America, where refugee populations face distinct sociopolitical and environmental challenges.

## 7 Implications for policy and practice

The differential patterns of mental health outcomes underscore the necessity for context-specific interventions. For camp residents, strategies should prioritize trauma-informed care, psychosocial stabilization, and enhancement of environmental safety. For community-dwelling refugees, interventions must focus on facilitating integration, addressing post-migration stressors, and promoting social and economic inclusion. Importantly, longitudinal monitoring is essential to capture the dynamic interplay between trauma exposure, environmental factors, and psychosocial adjustment over time. A successful intervention should involve the community directly considering the municipal and national levels, in each hosting country. Concerning the intervention team modality, intercultural and community efforts should be reunited to attend refugee youth with appropriate instruments, mainly tailored according to age, gender and prior background.
